# Professional Individuality

**Published:** 1863-04

**Authors:** Geo. F. Foote


### PROFESSIONAL INDIVIDUALITY.

BY GEO. F. FOOTE, M. D.

(Read before the Cincinnati Dental Association.)

Doubtless every dentist desires the advancement of his profession, and would heartily rejoice in its progressive elevation, yet how many of us recognize our own individual relation to it?

The sands upon the sea shore are made up of individual grains, the ocean by drops of water, each one of either is an essential of its whole, and there can be no bodies of sand without grains, or oceans without drops.

So in the profession each dentist is an essential of the whole, and as much of a component part thereof as any particular is of a general. He counts one, an individual of this grand whole we call the profession.

But while the grains of sand or drops of water are materially fixed in their individual and general relations, intelligence enables and designs the dentist to be progressive, and change not only his own individual relations, but that of the whole profession.

Without this progressive change he is as a mere grain of sand and of about the same use, having a physical and numerical existence only. Each one should feel an individual responsibility, and take upon his own shoulders the good and welfare of the whole—shoulders made broad by study and research, industry and perseverance. Every one should feel and govern himself as though he indivially were responsible for the whole, and in his researches, labor with reference to the whole. Every new fact or discovery, the result of diligent application and untiring zeal, should be a contribution to the profession for its general good, and his own advancement.

It is by contributions from those only who have this feeling, that the profession is advancing steadily toward the goal of greater excellence.

Each one can, from his own particular stand point, do something for the general good of all. He can reach out beyond the manor to which he was born, or primarily educated in, and turn over some stone yet unturned; and by diligence and honesty of purpose, he will be sure to find a gem, be it ever so small, that shall be of use in the profession. Let him cast this into the general coffer already rich with like contributions from other honest seekers after light and truth, and he will receive in exchange a diadem, filled with gems and pearls and precious stones of great price.

But it may be asked what shall an individual do to advance the science of dentistry? How shall he commence? Where shall he begin and in what direction shall he direct his efforts? This is truly an important question, for on it depends his own individual success, as well as his professional use and standing, and its answer is—Let him first inform himself of what is already known and published. This will save much time in useless search through fields already well beaten by the tread of other persons. Where the stones have been turned and overturned until their faces are familiar to every intelligent and well informed member of the profession. Let him commence at the A B C of Dentistry, as a science, and by diligent, persevering study, reading and association, familiarize himself with the record of both past and present.

Thus equipped he is prepared to render good service to his patients and himself, and at the same time he can levy contributions from the untrodden fields of science for the good and welfare of the whole.

This is the true course of every honorable high minded dentist. Let no one stop on a mere selfish plain, while from indolence he neglects to search for these valuable gems, or when obtained withholds them to inquire—"Will it pay?"

It always pays to do right—it always pays to do good, and he who casts his seed abroad with a generous hand, in due time receives a manifold return.

